# Case Report: Arthroscopic treatment of cedell fracture, shepherd fracture, and avulsion fracture at the insertion of the calcaneofibular ligament

**DOI:** 10.3389/fsurg.2025.1490126

**Published:** 2025-03-25

**Authors:** Sitong Zhang, Dejian Li, Qian Wang

**Affiliations:** Department of Orthopedics, Shanghai Pudong Hospital, Fudan University Pudong Medical Center, Shanghai, China

**Keywords:** talar fracture, cedell fracture, shepherd fracture, arthroscopy, internal fixation

## Abstract

Talar fractures represent less than 1% of all fractures, and combined fractures involving the posteromedial and posterolateral talar processes along with a lateral process fracture are exceptionally rare. These fractures are considered severe injuries that may lead to prolonged disability and persistent pain. The intricate anatomical configuration and the proximity of nerves and blood vessels surrounding the talus present substantial challenges in the management of posterior talar process fractures. Surgical procedures in this area are rare, and inadequate treatment may lead to significant discomfort and limitations in daily functioning for patients. In this case report, we describe a 45-year-old male who experienced a fall from a 2-meter height, leading to persistent right ankle swelling for 6 days. Radiographs, CT scans, and 3D-CT reconstructions identified fractures involving the posteromedial and posterolateral talar processes, as well as an avulsion fracture at the calcaneofibular ligament attachment site. To the best of our knowledge, there are no previously documented reports of this combined injury. We conducted arthroscopically assisted reduction and internal fixation through a posterior approach utilizing Herbert screw. The patient underwent a 4-year postoperative follow-up, during which favorable fracture healing was observed. The objective of this report is to demonstrate that arthroscopy offers a well-defined surgical field, aids in reduction and internal fixation, and to suggest a novel treatment approach for this uncommon fracture pattern.

## 1 Introduction

Fractures of the posterior talar process are documented to represent around 20% of all talar fractures. These fractures are commonly linked to sports-related injuries and, given the sometimes ambiguous injury mechanisms, they are frequently misinterpreted as ankle sprains or disregarded altogether. Therefore, it is imperative to maintain a high level of clinical suspicion, and specialized radiographic imaging or other diagnostic modalities may be necessary for accurate diagnosis (1). The posterior talar process is relatively large and consists of two tubercles: the lateral tubercle and the medial tubercle, which are divided by the groove for the flexor hallucis longus (FHL) tendon (2). The initial recorded instance of a posterior talar process fracture was delineated by Shepherd in 1882, and in 1974, Cedell documented a fracture specifically involving the medial tubercle, subsequently recognized as Cedell fracture (3, 4). Subsequent classifications have categorized posterior talar process fractures into medial tubercle fractures (Cedell fractures) and lateral tubercle fractures (Shepherd fractures). These fractures present notable diagnostic complexities, and inadequate attainment of anatomical reduction may result in post-traumatic arthritis or deformity (5). The uniqueness of this case lies in the fact that it is combined with a calcaneofibular ligament avulsion fracture. The calcaneofibular ligament (CFL) is commonly injured during ankle inversion, particularly when the foot is in a dorsiflexed position. This mechanism often occurs during sports activities, leading to lateral ankle instability. Recent studies highlight that CFL injuries are frequently associated with concomitant anterior talofibular ligament (ATFL) tears, exacerbating functional impairment (6). In terms of treatment, current approaches are primarily categorized into conservative and surgical options. Conservative treatment, such as casting or bracing, is typically the preferred choice for patients with small fracture fragments and stable joint integrity. However, for patients with larger fracture fragments, joint instability, or associated ligament tears, surgical intervention is generally more effective (7). In recent years, minimally invasive surgery assisted by arthroscopy has gained increasing favor due to its reduced trauma and improved recovery time (8). This technique enables precise reduction and internal fixation, effectively restoring the anatomical structure of the joint surface and minimizing postoperative complications (9). In this case report, we present a male patient with a combined Cedell and Shepherd fracture, in addition to an avulsion fracture at the calcaneofibular ligament attachment site. The patient underwent successful treatment with arthroscopically assisted minimally invasive reduction and internal fixation, resulting in a complete recovery.

## 2 Case presentation

**Figure 1:**
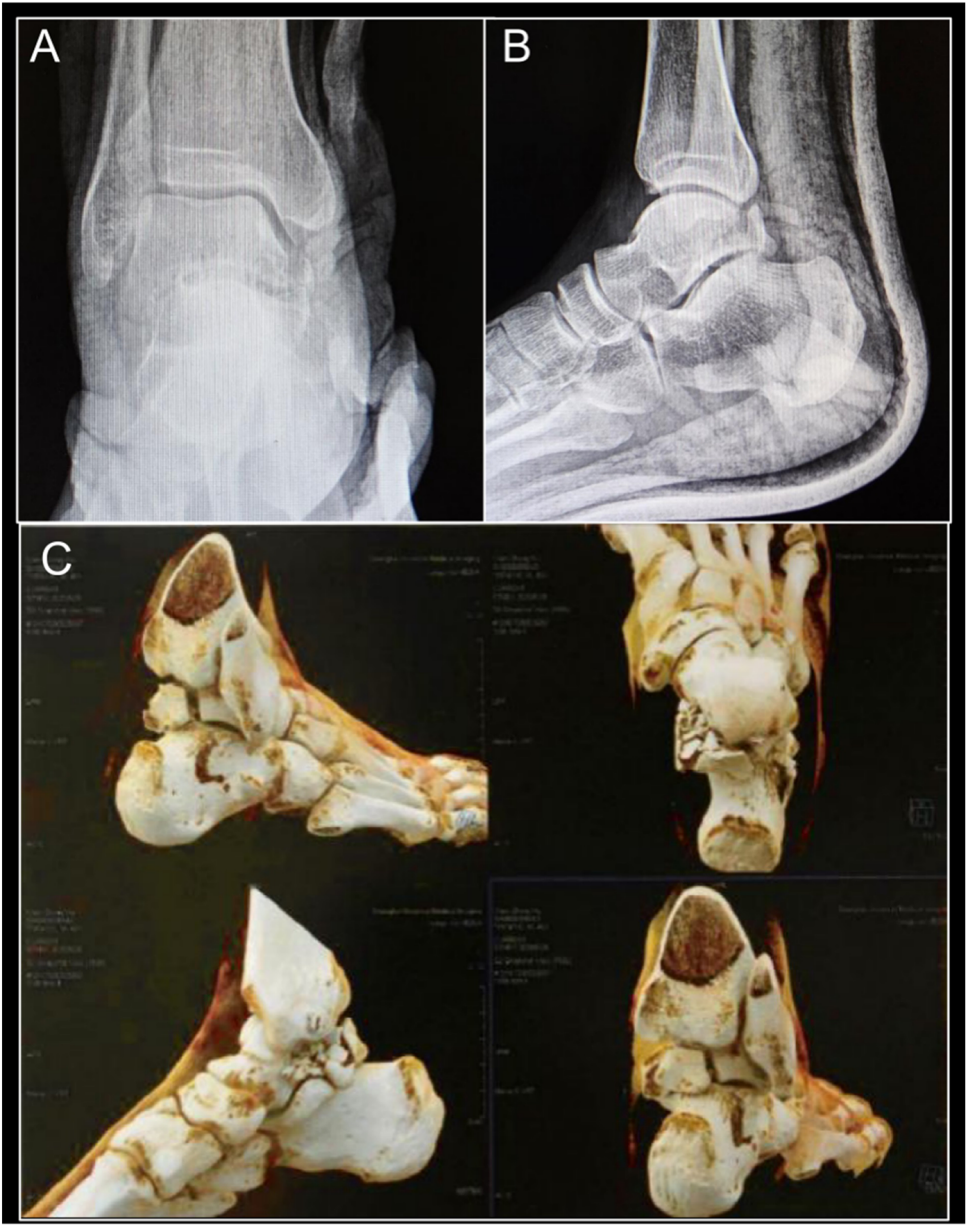
Anteroposterior **(A)** and lateral **(B)** x-rays showing a talar fracture; 3D-CT **(C)** scan showing fractures of the medial and lateral tubercles of the posterior talus and calcaneofibular ligament avulsion fracture.

A 45-year-old male patient presented with right ankle pain and swelling, along with limited mobility, persisting for six days subsequent to a fall from a 2-meter-high ladder. The patient had no prior history of surgery or trauma. He reported enduring pain in the right ankle. Upon physical examination, swelling and deformity of the right ankle were evident, without observable indications of vascular or neurological impairment. Due to significant swelling in the foot and ankle, there is severe limitation in inversion and eversion. Ankle range of motion is limited to dorsiflexion of 5° and plantarflexion of 10°. The patient has no history of hypertension, diabetes, cardiovascular diseases, osteoporosis, or hyperparathyroidism, and has no history of smoking. Anteroposterior and lateral radiographs of the right ankle indicated a fracture line in the posterior talar process and a possible fracture of the medial talar tubercle (Figures 1A,B). Subsequent 3D-CT imaging confirmed fractures involving the lateral and medial tubercles of the posterior talar process, in addition to an avulsion fracture at the calcaneal attachment of the calcaneofibular ligament(CFL) (Figure 1C). According to the AO classification of talar fractures, this case was categorized as type C2.2: comminuted fracture of the lateral tubercle of the posterior talar process.

**Figure 2:**
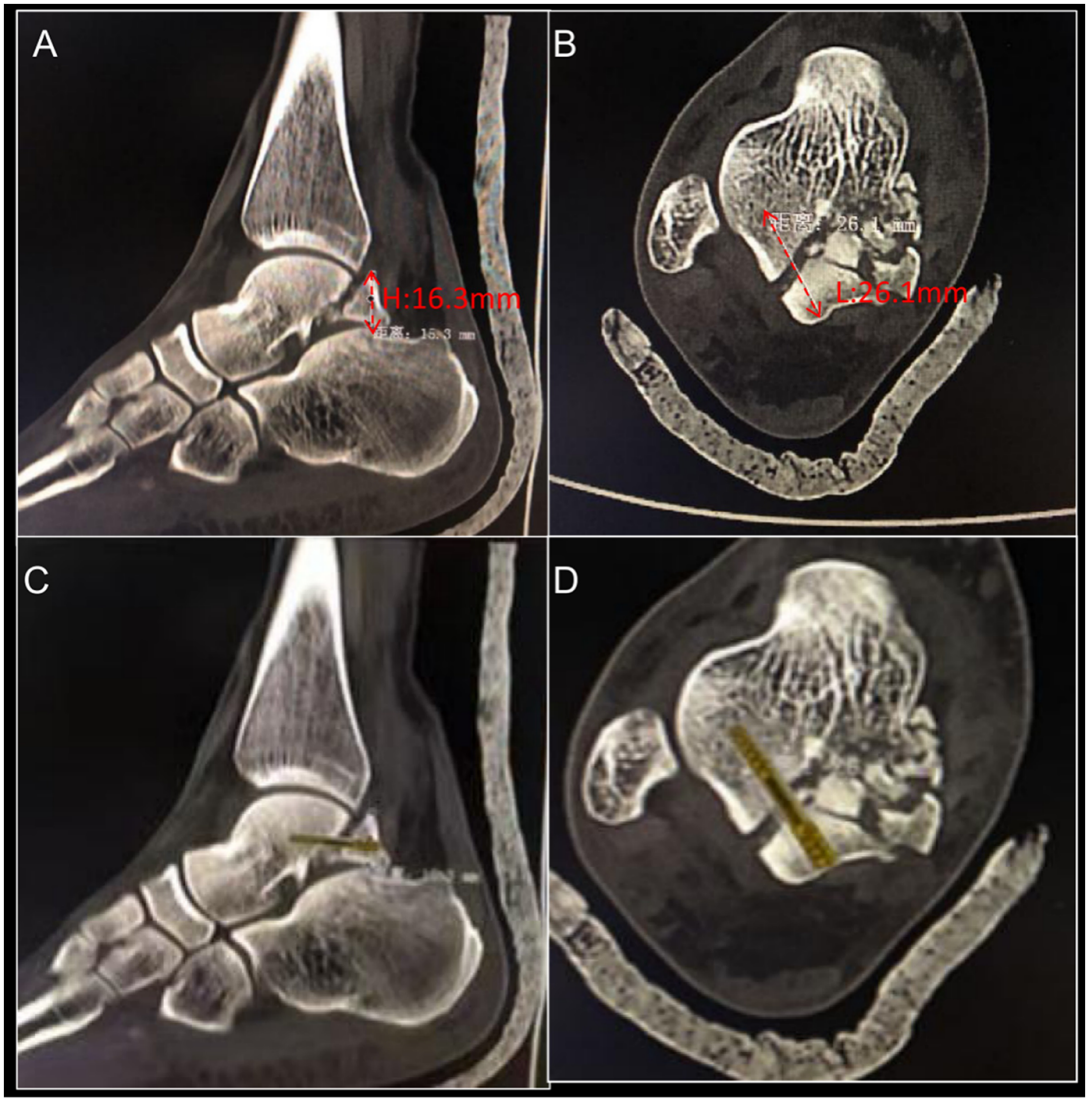
**(A)** CT sagittal view showing the fracture fragment with a height of 16.3 mm. **(B)** CT axial view showing the screw fixation length of 26.1 mm. CT sagittal view **(C)** and axial view **(D)** with fitted Herbert screw, determining the screw length and surgical plan.

**Figure 3:**
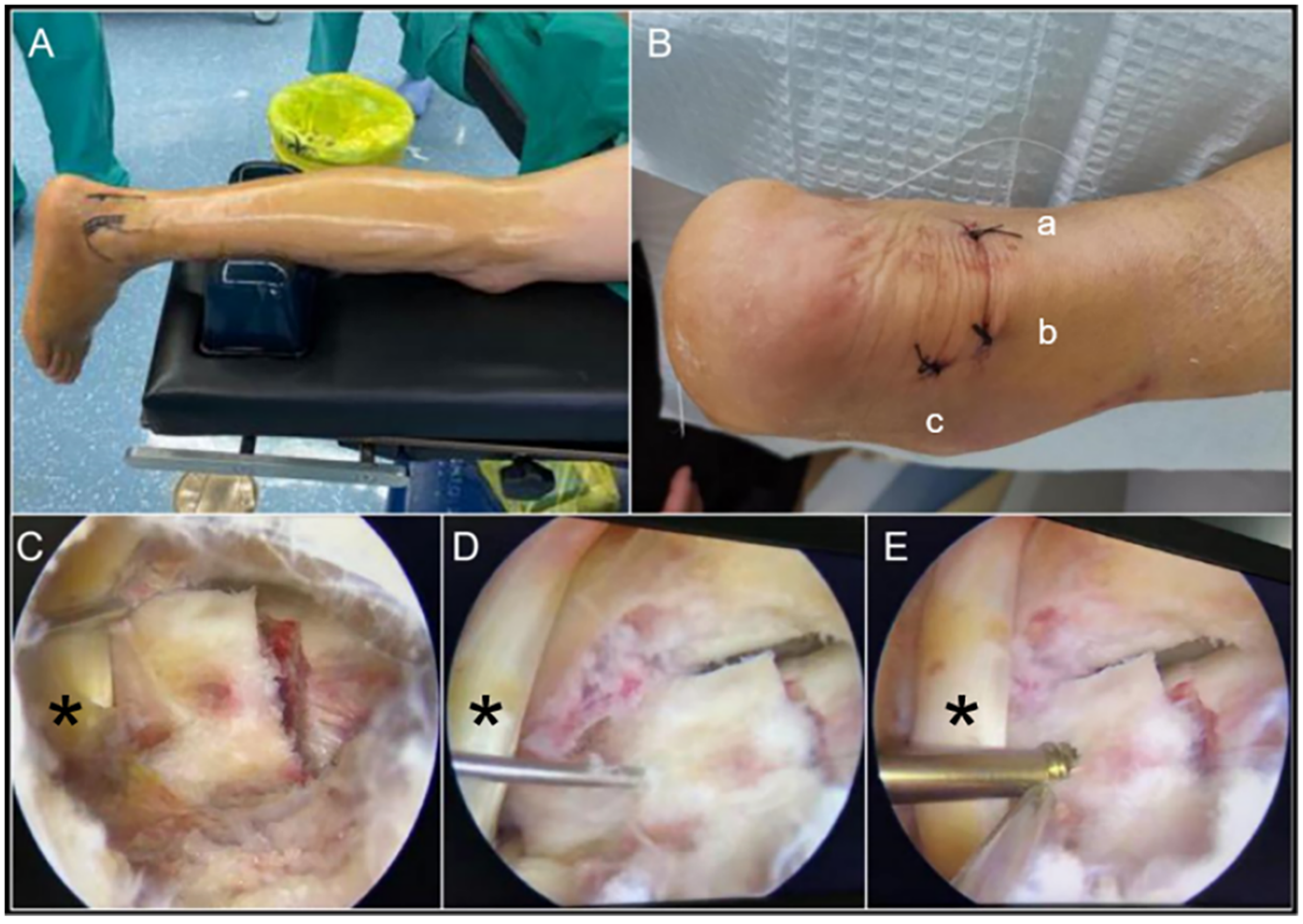
**(A)** Arthroscopically assisted surgery with the patient in the prone position and a silicone pillow placed under the lower leg. **(B)** Three surgical incisions used for arthroscopically assisted reduction and internal fixation **(a)** posteromedial approach; **(b)** posterolateral approach; **(c)** assisted posterolateral approach). **(C)** The arthroscopic probe was used to laterally retract the FHL, exposing the fracture site, via a posteromedial approach under arthroscopy **(D)** Arthroscopic fracture reduction followed by K-wire fixation. **(E)** Internal fixation with Herbert screw assisted by arthroscopy. (*: FHL, flexor hallucis longus tendon).

Upon literature review, it was noted that Cedell and Shepherd fractures are uncommon, typically managed with open reduction and internal fixation using a posterior approach. However, considering the severity of the lateral tubercle fracture and the minimal displacement of the calcaneofibular ligament avulsion fracture in this patient, a less invasive approach was chosen to reduce trauma and expedite the surgical procedure. After discussion, surgical planning (Figures 2A,B), and simulation (Figures 2C,D), it was determined that arthroscopically assisted reduction and internal fixation of the medial tubercle of the posterior talar process would be conducted with the patient positioned in a prone orientation (Figure 3A).

The procedure was performed employing a three-incision approach adjacent to the Achilles tendon (Figure 3B). One approach was utilized for arthroscope insertion to visualize the fracture site, another for the insertion of instruments such as an arthroscopic shaver and screw, and the third for employing an arthroscopic probe to assist in the reduction process. Throughout the procedure, meticulous attention was given to identifying and safeguarding the flexor hallucis longus tendon and the neurovascular bundle within the tarsal tunnel. Subsequent to exposing the fracture of the posterior talar process (Figure 3C), debridement of the fracture site was performed utilizing an arthroscopic shaver to eliminate fragmented tissue and small loose bone fragments, followed by reduction achieved with the use of an arthroscopic probe (Figure 3D). Temporary fixation was achieved using Kirschner wires, followed by the sequential insertion of Herbert screw with a diameter of 3 mm and a length of 24 mm for the definitive fixation of the medial tubercle of the posterior talar process (Figure 3E). Intraoperative fluoroscopy was utilized to verify the accurate placement of the screws. Given the comminuted Shepherd fracture and the minimal displacement observed in the calcaneofibular ligament avulsion fracture, no additional intervention was considered necessary.

**Figure 4:**
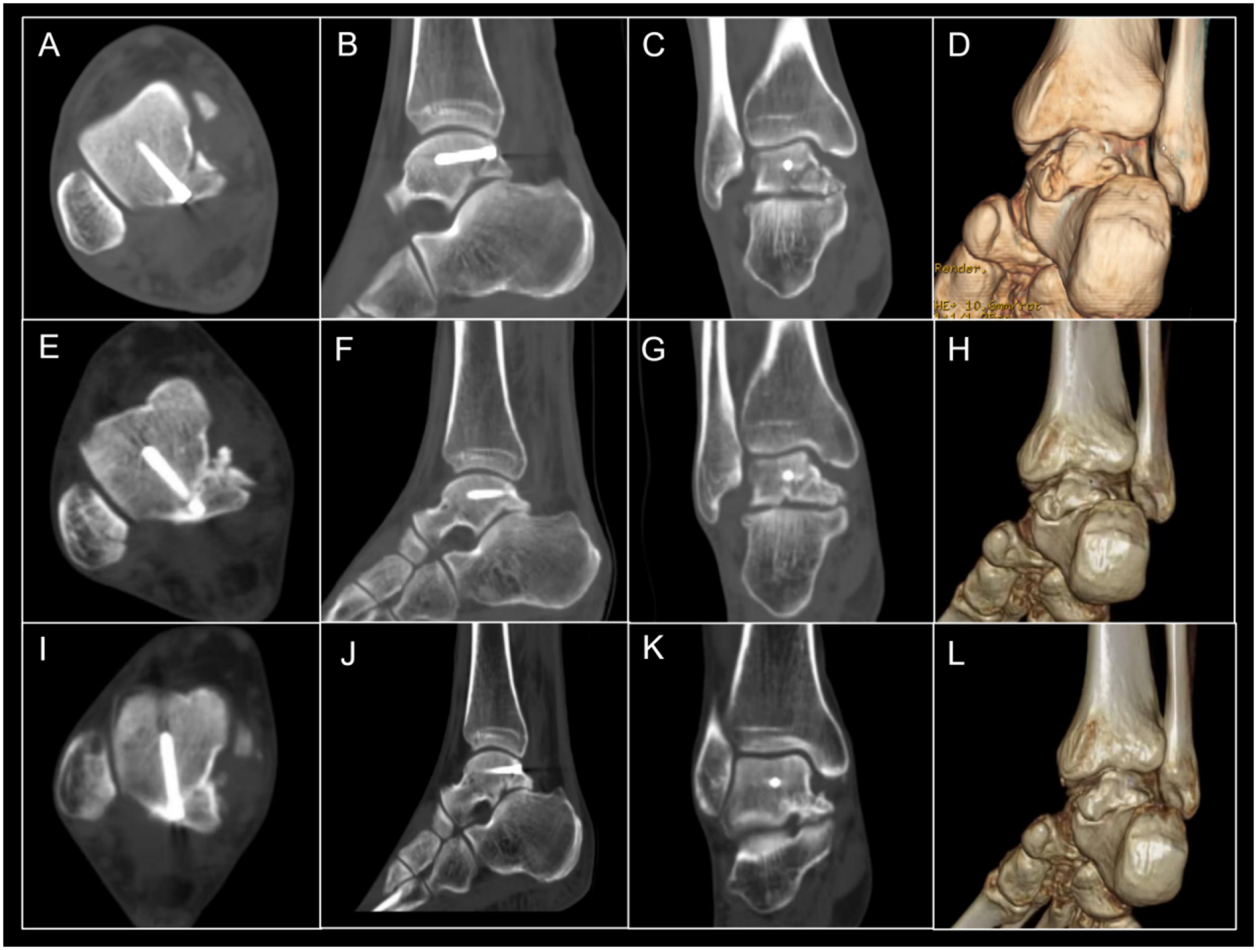
CT scan **(A–D)** 2 days postoperatively showing good fixation; CT scan **(E–H)** 2 months postoperatively showing signs of healing in the fixed fracture fragment; CT scan **(I–L)** 2 years postoperatively showing favourite fracture healing, with improved healing of the posterior lateral tubercle.

Postoperatively, the patient was instructed to undergo routine rehabilitation and follow-up. In the course of postoperative rehabilitation, patients were assessed using the Visual Analog Scale (VAS) and the American Orthopaedic Foot and Ankle Society (AOFAS) Ankle-Hindfoot Scale. VAS scores indicated that the patient reported a pain level of 4 on postoperative day 2, 3 at 2 months, 2 at 1 year, and 0 at both 2 and 4 years, reflecting a progressive reduction in pain over time, ultimately returning to baseline. Regarding the AOFAS scores, the patient achieved a score of 40 on postoperative day 2, 66 at 2 months, 86 at 1 year, and 96 at both 2 and 4 years, illustrating a steady recovery of foot and ankle function, ultimately reaching a satisfactory level. A CT scan conducted 2 days post-surgery revealed adequate fixation of the fracture (Figure 4A [sagittal view], Figure 4B [coronal view], Figure 4C [axial view], Figure 4D [3D-CT]). At 2 months, CT imaging indicated early signs of healing at the fracture site of the medial tubercle fixed with screw (Figure 4E [sagittal view], Figure 4F [coronal view], Figure 4G [axial view], Figure 4H [3D-CT]). A CT scan at 2 years postoperatively demonstrated complete fracture healing (Figure 4I [sagittal view], Figure 4J [coronal view], Figure 4K [axial view], Figure 4L [3D-CT]). The patient went back to work and was highly satisfied.

## 3 Discussion

The talus is a unique and complex bone in the foot, playing a critical role in the ankle joint's function. The body of the talus has a distinctive shape that allows it to fit snugly within the ankle mortise formed by the tibia and fibula. Its upper part is wider compared to the lower part, which is narrower. Similarly, the front of the talus is wider than the back, creating a wedge-shaped structure that contributes to the stability of the ankle joint (10). Extending posteriorly, the talus forms an arc that leads to a slender posterior process. This posterior process is further divided into two tubercles: the medial and lateral tubercles. These tubercles are important as they create a groove between them, which is the path for the FHL tendon. This tendon runs along this groove, playing a vital role in the movement of the hallux and contributing to the overall mechanics of the foot. The anatomical features of the talus are essential for its function in weight-bearing and movement, making it a key bone in the lower limb. The medial tubercle is the attachment point of the posterior tibiotalar ligament, and the lateral tubercle is the attachment point of the posterior talofibular ligament (2). Fractures of the posterior talar process are infrequent, posing diagnostic challenges. These fractures are typically categorized by location as Cedell fractures (involving the medial tubercle) or Shepherd fractures (involving the lateral tubercle), with the combination of Cedell and Shepherd fractures being exceptionally rare, which increases the risk of misdiagnosis or missed diagnosis (3, 4). Timely reduction and surgical management are imperative for posterior talar process fractures (2); failure to achieve anatomical reduction may lead to chronic pain, malunion, or post-traumatic arthritis, significantly affecting the patient's quality of life (5, 11). The injury mechanism in combined Cedell and Shepherd fractures, involving the entire posterior talar process, remains unclear. Nasse and Manol suggest that excessive plantarflexion of the ankle joint results in compression between the posterior tibia and calcaneus, culminating in fractures of the posterior talar process (12). Ebraheim etal reported that in cases where the ankle is in plantarflexion and the foot is in a varus position, the sustentaculum tali of the calcaneus exerts an impact on the posterior medial tubercle of the talus, resulting in its fracture. Concurrently, hyperextension of the ankle joint increases the tension on the posterior talofibular ligament, leading to an avulsion fracture of the posterior lateral tubercle of the talus (13, 14). The ankle joint is subjected to direct force in plantar flexion, and the posterior process of the talus sustains a ‘nutcracker’ injury between the posterior malleolus and the calcaneus, resulting in a lateral posterior tubercle fracture (10). In this particular case, we postulate that the patient incurred a combined Cedell and Shepherd fracture, along with an avulsion fracture at the attachment site, as a consequence of pronounced plantarflexion, adduction, and internal rotation of the foot subsequent to a fall from an elevated position. Based on the AO classification of talar fractures, this case was categorized as type C2.2. A comprehensive review of the literature yielded no prior references or documented reports pertaining to the management of such intricate fractures.

Conventionally, fractures of the posterior talar process are managed through open reduction and internal fixation, a surgical intervention linked to considerable patient trauma and an elevated risk of postoperative complications (15). In a study with an average follow-up of 7.5 years involving 20 patients who underwent surgery for talar fractures, 4 patients (20%) developed avascular necrosis of the talus, 7 patients (35%) required revision surgery, and radiographic analysis revealed a 94% incidence of osteoarthritis (16). In a separate retrospective study encompassing 50 cases of talar body fractures treated with open reduction and internal fixation, a 40% occurrence of osteonecrosis was documented, along with tibiotalar and subtalar joint arthritis rates of 65% and 35%, respectively, at the three-year postoperative mark (17). In the management of posterior talus fractures, while open surgery provides clear operative visibility, complex fractures often require medial malleolar osteotomy for adequate exposure. However, increasing the risk of complications, including nonunion, malunion, delayed union, chronic pain, and post-traumatic arthritis at the osteotomy region. In contrast, arthroscopic surgery, as a minimally invasive technique, avoids the need for medial malleolar osteotomy while providing clear visualization of the fracture zone, thus reducing surgical trauma. Nevertheless, posterior ankle arthroscopy requires advanced expertise, particularly in the proficient execution of posterior ankle arthroscopic techniques. Successfully performing this procedure requires a solid foundation in basic posterior ankle arthroscopic skills. Notably, this complex surgery was performed without the need for specialized equipment; instead, conventional instruments were employed, facilitated by careful preoperative planning and measurements (Figure 2), which ultimately contributed to favorable clinical outcomes. From a technical perspective, it is essential for the surgeon to have a precise understanding of the location of the tibial nerve and tibial vessels. In our approach, the FHL is first exposed and serves as a reference point. All instruments are then carefully maneuvered to avoid crossing the medial side of the tendon. Additionally, in posterior talus fractures, the fracture fragments are typically small, so the surgeon must ensure successful screw placement on the first attempt and avoid excessive force during screw insertion to prevent fragment fragmentation. Repeated manipulation can lead to further fracture of the bone fragments, which may compromise the surgical outcome. Therefore, these procedures demand not only expertise in arthroscopic techniques but also significant clinical experience in managing traumatic fractures. This surgical approach is multifaceted and requires meticulous preoperative planning to minimize the risk of failure. Surgical failure can result in adverse outcomes, including post-traumatic arthritis, intra-articular loose bodies, avascular necrosis of the talus (7).

These findings indicate that although open surgery offers improved visibility, it may not consistently result in satisfactory anatomical reduction, thereby increasing the likelihood of various postoperative complications. Recently, with advancements in arthroscopic techniques, there have been documented cases of successful fracture reduction and fixation facilitated by arthroscopic guidance (15, 18). Arthroscopically assisted reduction and fixation not only minimize patient trauma and reduce the risk of wound infection but also allow for improved visualization of the fracture site, thereby contributing to improved surgical outcomes (19). In our case, given the severity of the lateral tubercle injury and the minimal displacement of the calcaneofibular ligament avulsion, we selected arthroscopically assisted reduction and internal fixation of the medial tubercle, resulting in favorable outcomes with reduced patient trauma. Regarding the surgical approach, posterior-lateral or posterior-medial approaches are typically chosen for treating talar fractures (5, 11, 20). In this case, the patient was positioned prone, and three incisions were made to access the fracture site through both the posterior-lateral and posterior-medial approaches under arthroscopic guidance. Postoperative rehabilitation plays a critical role in preventing complications associated with foot and ankle fractures. Early weight-bearing and functional exercises have been shown to facilitate the restoration of joint range of motion, reduce stiffness, and mitigate muscle atrophy. Research indicates that early weight-bearing (initiated as early as two weeks post-surgery) does not increase the risk of complications compared to delayed weight-bearing and may contribute to improved overall patient health. Furthermore, postoperative rehabilitation should incorporate physical therapy, with a particular focus on enhancing ankle joint mobility and strengthening, in order to optimize functional recovery (21).

Ultimately, the arthroscopically assisted reduction and internal fixation resulted in successful fracture healing. In this study, we present a case of posterior talus fracture treated through meticulous preoperative planning and innovative surgical techniques. Given that this approach was applied to a single case, the generalizability and persuasiveness of the conclusions may be limited. In the future, we aim to extend this surgical technique to a larger cohort of talus fracture patients in order to accumulate more case data, thereby providing stronger evidence-based support for the efficacy and feasibility of this surgical approach.

## 4 Conclusion

The occurrence of combined Cedell and Shepherd fractures involving the posterior talar process is exceptionally uncommon, and the presence of an avulsion fracture at the CFL attachment further adds to the rarity of the case. These complex injuries pose a risk of misdiagnosis or delayed diagnosis, underscoring the necessity for prompt recognition and intervention, emphasizing the critical significance of early evaluation and treatment strategizing. In our case, arthroscopically assisted reduction and fixation via a posterior approach yielded excellent outcomes. As arthroscopic techniques continue to advance, their application in managing complex intra-articular hindfoot fractures is anticipated to become increasingly common in clinical practice.

## Data Availability

The original contributions presented in the study are included in the article/Supplementary Material, further inquiries can be directed to the corresponding authors.
